# A Switched Approach for Smartphone-Based Pedestrian Navigation

**DOI:** 10.3390/s24165247

**Published:** 2024-08-14

**Authors:** Shenglun Yi, Mattia Zorzi, Xuebo Jin, Tingli Su

**Affiliations:** 1 Department of Information Engineering, University of Padova, Via Gradenigo 6/B, 35131 Padova, Italy; shenglun@dei.unipd.it; 2 School of Artificial Intelligence, Beijing Technology and Business University, Beijing 100048, China; jinxuebo@btbu.edu.cn (X.J.); sutingli@btbu.edu.cn (T.S.)

**Keywords:** pedestrian navigation, adaptive Kalman filtering, bias estimation

## Abstract

In this paper, we propose a novel switched approach to perform smartphone-based pedestrian navigation tasks even in scenarios where GNSS signals are unavailable. Specifically, when GNSS signals are available, the proposed approach estimates both the position and the average bias affecting the measurements from the accelerometers. This average bias is then utilized to denoise the accelerometer data when GNSS signals are unavailable. We test the effectiveness of denoising the acceleration measurements through the estimated average bias by a synthetic example. The effectiveness of the proposed approach is then validated through a real experiment which is conducted along a pre-planned 150 m path.

## 1. Introduction

Smartphone-based pedestrian navigation systems (PNSs) are significant tools for various human activities, including healthcare monitoring [1,2,3], location-based services (LBSs) [4,5,6], and tourism management [7,8,9]. Generally, the primary technology available for PNS is the Global Navigation Satellite System (GNSS), typically embedded in our smartphones, which can provide continuous and relatively accurate location information, including long-term operations in outdoor environments [10,11,12,13]. Furthermore, with advancements in GNSS technology, services offering differential correction techniques for GNSS measurements (some of which are free) are routinely used to obtain position estimates whose accuracy is at the meter level [14,15]. However, in challenging environments such as urban areas, canyons, tunnels, and indoors, the accuracy of GNSS signals may be degraded or interrupted [16,17,18,19,20,21,22,23]. To address this problem, one option is to utilize 3D-map-aided pedestrian positioning tools that have been previously developed to correct the GNSS signals or mitigate their unavailability [24,25,26]. However, the creation and use of 3D city maps can be costly (in economic and computational terms). Another option is to combine multiple infrastructures such as WiFi, Ultra-Wideband (UWB), and optical tracking systems (OTSs) to enhance the accuracy of position estimates in a complementary manner [24,27,28,29,30,31,32,33,34,35]. However, in urban areas characterized by dense buildings, tunnels, or overpasses, smartphones typically can only receive continuous and stable signals from “sourceless” systems, specifically an IMU manufactured with low-cost micro-electromechanical system (MEMS) technology [36,37,38].

In such situations, IMU-based pedestrian navigation systems are unique devices that can provide information about the pedestrian position by means of strapdown integration algorithms (SAs) [39,40,41]. However, the error in estimating the pedestrian position using only IMU signals tends to increase over time primarily due to biases in accelerometers which manifest as constant offsets. Even small biases, combined with small sensor measurement noises from the accelerometers, accumulate over time during integration operations, leading to severe errors in velocity and position estimates. Numerous studies have been conducted to address this issue in IMUs. One well-known solution to this problem is the pedestrian dead reckoning (PDR) method [4,42,43,44]. The latter exploits the zero-velocity updating (ZUPT) technique [16,45,46,47,48], which leverages the observation that foot speed should be zero when the foot is in contact with the ground during walking. This approach helps to mitigate errors that occur due to the bias in the measurements of the accelerations. However, a limitation of ZUPT regards the strict requirements on sensor placement: the IMU should be placed on the feet of the pedestrian, i.e., an impractical solution with the sole use of the smartphone. Alternatively, one can use learning-based methods, such as human motion pattern recognition [49,50,51,52,53]. The recent trade is to use artificial intelligence (AI)-based algorithms [54,55,56,57,58] to compensate for measurement outages, i.e., when the GPS signal is unreliable. Empirical studies show that AI-based algorithms can predict GPS pseudo increments through online learning. The main limitation of this second solution is that these methods are computationally expensive, and as a consequence, the execution of these algorithms on a smartphone causes a rapid discharge of the battery.

The aim of this paper is to propose a switched approach to perform smartphone-based pedestrian navigation tasks, even in scenarios where GNSS signals are unavailable, without using algorithms whose computational cost is expensive or requiring invasive sensors. The proposed approach computes the estimate of the pedestrian position in two different ways switching from one to the other depending on the availability of the GNSS signals. When the GNSS signals are available, the procedure estimates the pedestrian position and the bias affecting the measurements coming from the accelerometers by means of an adaptive Kalman filter. This bias is averaged over a time window in order to prevent occasional inaccurate estimates in some specific time steps. When the GNSS signals are unavailable, the accelerometer signals are denoised through the average bias previously estimated. Then, the pedestrian position is estimated using an adaptive Kalman filter. The experiments showed that the estimated average bias contains useful information that can be exploited when the GNSS is not available. Therefore, we envision that the estimated average bias could be incorporated in the PDR technology, which relies on acceleration measurements coming from the IMU device, in order to improve the so-called “PDR pedestrian step estimation” task.

The outline of this paper is as follows. In Section 2, we introduce the switched approach for smartphone-based pedestrian navigation tasks. In Section 3.1, we test, through a synthetic example, the validity of denoising the acceleration measurements through the estimated average bias. In Section 3.2, we validate the proposed approach through a real experiment which is conducted along a pre-planned 150 m path and show that in both a GNSS-free environment and GNSS-denied environment, the Root Mean Square Error of the estimated pedestrian position is always less than 1 m. Finally, in Section 4, we draw the conclusions.

## 2. The Proposed Approach

Consider a pedestrian having a smartphone equipped with both the exteroceptive sensor (GNSS) and the proprioceptive sensor (IMU), which comprises an accelerometer and a rate gyro. We aim to address the following 2D pedestrian navigation problem: let $p_{k}={\left[\;p_{N,k}\;p_{E,k}\;\right]}^{⊤}\in R^{2}$ [m] denote the position of the pedestrian relative to the east–north–up coordinates system (ENU-system) at time *k*; given the available data at time *k* from the smartphone sensors (i.e., GNSS and IMU), we want to compute an estimate, say $p_{k|k}$ [m], of $p_{k}$.

In the case where the GNSS signals are available, the accuracy of the estimate $p_{k|k}$ is generally satisfactory. However, in obstacle-dense environments, such as indoors, under dense tree cover, or in urban canyon, GNSS signals often degrade or disappear entirely, and the sole onboard IMU signals do not provide enough information to obtain a reliable estimate of the pedestrian position due to its cumulative error mainly caused by the constant bias affecting the accelerometers. As a consequence, the resulting estimate $p_{k|k}$ based solely on the IMU signals will not be accurate enough.

**Figure 1:**
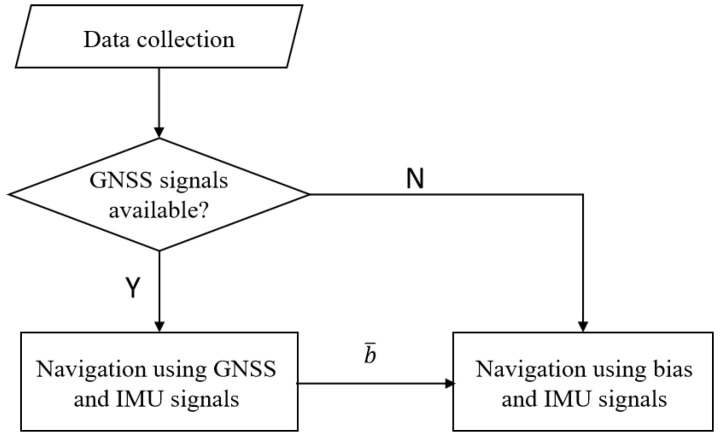
The switched approach for pedestrian navigation.

In what follows, we propose a switched approach to compute $p_{k|k}$: the estimation is performed in two different ways depending on whether the GNSS signals are available or not. In the case where the GNSS signals are available (i.e., we perform navigation using GNSS signals), we exploit the GNSS and IMU data to estimate $p_{k}$ and the bias on accelerometers. In order to obtain a robust estimate of the bias, we compute its average over a time window of length *N*, and the latter is denoted by $\bar{b}$ [m/s^2^]. In the case where the GNSS signals are unavailable (i.e., we perform navigation without GNSS signals), the estimated average bias, computed when the GNSS signals were available, is used to denoise the signals obtained from the accelerometers. Using the “denoised” IMU data, we compute an accurate estimate of $p_{k}$. The switched scheme we propose is illustrated in Figure 1.

In what follows, we describe in detail the navigation tasks with and without navigation signals. In order to streamline the presentation of these two tasks, we assume that the time instant in which the switch happens is k=1 for both the tasks.

### 2.1. Navigation Using GNSS Signals

The sensors available (i.e., able to provide information about the pedestrian position) in the smartphone are as follows:

- An inertial measurement unit whose axes are aligned with the principal axes of the smartphone. The latter comprises two types of triaxial sensors that provide the measurements expressed in the local coordinate system (L-system): an accelerometer that measures the specific force $a_{m,k}\in R^{3}$ [m/s^2^], and a rate gyro that measures the angular velocity $w_{m,k}={\left[\;\phi_{k}/T\;\theta_{k}/T\;\psi_{k}/T\;\right]}^{⊤}\in R^{3}$ [rad/s], where *T* is the IMU sampling time, $\phi_{k}$ [rad] is the roll angle, $\theta_{k}$ is the pitch angle, and $\psi_{k}$ [rad] is the yaw angle.
- A GNSS receiver that gathers the position measurements $p_{m,k}={\left[\;p_{mN,k}\;p_{mE,k}\;\right]}^{⊤}\in R^{2}$ [m] as well as the corresponding velocities $v_{m,k}={\left[\;v_{mN,k}\;v_{mE,k}\;\right]}^{⊤}\in R^{2}$ [m/s] both expressed in the ENU-system.

The dynamic of the pedestrian is described by the following inertial-aided model [59]:

(1) $$x_{k+1}=Ax_{k}+Ba_{G,k}+\varepsilon_{k}$$

where

$$A=\left[\begin{array}{ccc} I_{2} & TI_{2} & 0\\ 0 & I_{2} & 0\\ 0 & 0 & I_{3} \end{array}\right]\in R^{7\times 7},\;\;B=\left[\begin{array}{cc} 0.5T^{2}I_{2} & 0\\ TI_{2} & 0\\ 0 & 0 \end{array}\right]\in R^{7\times 3},$$

$I_{n}\in R^{n\times n}$ is the identity matrix; $x_{k}={\left[\;p_{k}^{⊤}\;v_{k}^{⊤}\;b_{k}^{⊤}\;\right]}^{⊤}$ is the state, in which $p_{k}\in R^{2}$ [m] and $v_{k}\in R^{2}$ [m/s] are the position vector and the velocity vector at time *k* of the pedestrian in the ENU-system, and $b_{k}\in R^{3}$ [m/s^2^] is the vector bias on accelerometers in the L-system. Moreover, $a_{G,k}$ is the global acceleration in the ENU-system:

(2) $$a_{G,k}=M_{k}\left(a_{m,k}-b_{k}\right)+g_{N}$$

where

(3) $$M_{k}=M_{\phi_{k}}M_{\theta_{k}}M_{\psi_{k}}$$

is the rotation matrix representing the orientation of the L-system with respect to the ENU-system:

$$\begin{array}{c} M_{\phi_{k}}=\left[\begin{array}{ccc} \cos\phi_{k}, & 0, & \sin\phi_{k}\\ 0, & 1, & 0\\ -\sin\phi_{k}, & 0, & \cos\phi_{k} \end{array}\right],\;\;M_{\theta_{k}}=\left[\begin{array}{ccc} 1, & 0, & 0\\ 0, & -\cos\theta_{k}, & \sin\theta_{k}\\ 0, & \sin\theta_{k}, & \cos\theta_{k} \end{array}\right],\;\;M_{\psi_{k}}=\left[\begin{array}{ccc} \cos\psi_{k}, & \sin\psi_{k}, & 0\\ -\sin\psi_{k}, & \cos\psi_{k}, & 0\\ 0, & 0, & 1 \end{array}\right], \end{array}$$

and $g_{N}$ is the constant gravity vector in the ENU-system. Finally, $\varepsilon_{k}\in R^{7}$ is white Gaussian noise with unknown mean $q_{k}$ and unknown covariance matrix $Q_{k}$. It is not difficult to see that the dynamic model (1) can be expressed as follows:

(4) $$x_{k+1}=\Psi_{k}x_{k}+Bu_{k}+\varepsilon_{k}$$

where

(5) $$\Psi_{k}=A-BM_{k}\left[\;0\;0\;I_{3}\;\right],$$

and

(6) $$u_{k}=M_{k}\left(a_{m,k}+g_{N}\right).$$

The measurement model is defined as follows:

(7) $$y_{k}=Cx_{k}+\epsilon_{k}$$

where

$$C=\left[\begin{array}{ccc} I_{2} & 0 & 0\\ 0 & I_{2} & 0 \end{array}\right],$$

$y_{k}={\left[\;p_{m,k}\;v_{m,k}\;\right]}^{⊤}$ and $\epsilon_{k}\in R^{4}$ is the white Gaussian noise with unknown mean $r_{k}$ and unknown covariance matrix $R_{k}$.

Then, at time *k*, an estimate of the position of the pedestrian in the ENU-system, i.e., $p_{k}$, and the vector bias $b_{k}$ on the accelerometers in the L-system can be obtained from the state estimate $x_{k|k}$ of $x_{k}$ of the state space model (4)–(7). However, there is a main issue to address, that is, $q_{k},r_{k}$ and $Q_{k},R_{k}$ are unknown. This latter is addressed by using the adaptive Kalman filter [60,61,62,63] which computes both $x_{k|k}$ and the parameters characterizing the noise processes. The resulting algorithm at time k=1,2,… is the following:

1. Available information:
  $$y_{k},\;x_{k-1|k-1},\;u_{k-1},\;w_{m,k},\;a_{m,k},\;P_{k-1|k-1},\;q_{k-1},\;r_{k-1},\;Q_{k-1},\;R_{k-1}.$$
2. Prediction step:
  (8) $$x_{k|k-1}=\Psi_{k-1}x_{k-1|k-1}+Bu_{k-1}+q_{k-1}$$
  (9) $$P_{k|k-1}=\Psi_{k-1}P_{k-1|k-1}\Psi_{k-1}^{⊤}+Q_{k-1}.$$
3. Measurement noise parameter update:
  (10) $$r_{k}=\left(1-\eta_{k}\right)r_{k-1}+\eta_{k}\left(y_{k}-Cx_{k|k-1}\right)$$
  (11) $$e_{k}=y_{k}-Cx_{k|k-1}-r_{k}$$
  (12) $$R_{k}=\left(1-\eta_{k}\right)R_{k-1}+\eta_{k}\left(e_{k}e_{k}^{⊤}-CP_{k|k-1}C^{⊤}\right)$$
  where $e_{k}$ is the innovation, and $\eta_{k}=\left(1-\rho \right)/\left(1-\rho^{k}\right)$ and ρ∈[0,1] is the forgetting factor.
4. Update step:
  (13) $$K_{k}=P_{k|k-1}C^{⊤}{\left(CP_{k|k-1}C^{⊤}+R_{k}\right)}^{⊤}$$
  (14) $$x_{k|k}=x_{k|k-1}+K_{k}\left(y_{k}-Cx_{k|k-1}-r_{k}\right)$$
  (15) $$P_{k|k}=\left(I-K_{k}C\right)P_{k|k-1}.$$
5. Process noise parameters update:
  (16) $$q_{k}=\left(1-\eta_{k}\right)q_{k-1}+\eta_{k}\left(x_{k|k}-\Psi_{k-1}x_{k|k-1}\right)$$
  (17) $$\begin{array}{c} Q_{k}=\left(1-\eta_{k}\right)Q_{k-1}+\eta_{k}\left(K_{k}e_{k}e_{k}^{⊤}K_{k}^{⊤}+P_{k|k}-\Psi_{k-1}P_{k|k}\Psi_{k-1}^{⊤}\right). \end{array}$$
6. Compute
  $$\begin{array}{cc} p_{k|k} & =\left[\;I\;0\;0\;\right]x_{k|k}\\ b_{k|k} & =\left[\;0\;0\;I\;\right]x_{k|k}. \end{array}$$
7. Compute the average value of the bias vector over a window of length *N*
  $$\bar{b}=\frac{1}{N}\sum_{i=k-N+1}^{k}b_{i|i}.$$

It is worth noting that the estimate of the average bias is $\bar{b}$, i.e., the one computed in Step 7. This averaging is performed in order to prevent occasional inaccurate estimates in some specific time steps. Notice that, for the transient steps, i.e., *k* such that 1<k<N, we have $\bar{b}=\frac{1}{k}\sum_{i=1}^{k}b_{i|i}$.

**Remark** **1.** *It is worth noting that the performance of the adaptive Kalman filter depends on how accurate the state space model (4)–(7) is. In the case where the latter is not so accurate, e.g., when the sampling time T is not sufficiently small or the estimated covariance matrices are not so accurate, one could design an adaptive robust Kalman filter on the basis of the recent literature about robust Kalman filtering [64,65,66,67]. These approaches postulate that the actual model belongs to an ambiguity set which is a ball about the nominal model (i.e., (4)–(7)) in the topology induced by the Kulback–Leibler divergence. Its radius depends on the degree of accuracy of the nominal model. Moreover, these filters can also be generalized to ambiguity sets which are balls defined using more general topologies, see [68,69]. The appealing feature of these robust filters is that they exhibit convergence properties in the case of constant parameters [70,71,72], and they can be efficiently implemented since they have the same structure of the Kalman filter [73,74].*

### 2.2. Navigation without GNSS Signals

In this scenario, the GNSS signals are unavailable, and the only source of information comes from the onboard IMU. However, the error in the estimation of the pedestrian position using only the IMU signals tends to increase over time. This error is due to the so-called integration drift, i.e., the error generated by the double integration of $a_{m,k}$: Even small errors or biases in the measurements accumulate over time during integration, leading to increasing errors in velocity and position estimates. It is worth noting that the drift integration can be avoided by means of the pedestrian dead reckoning (PDR) technology [4]. However, the latter needs to estimate the number of steps during the walking of the pedestrian. Such information requires the use of computationally expensive algorithms or invasive sensors (e.g., put some sensors on the feet of the pedestrian).

In order to overcome the aforementioned limitations we address the issue regarding the integration drift using the average estimated bias in the L-system computed when the GNSS signals were available, i.e., $\bar{b}$. More precisely, we define the denoised measurement:

(18) $$y_{k}:=a_{m,k}-\bar{b}.$$

Then, we consider the state space model (called “current” statistical model; see, [75,76]):

(19) $$\begin{array}{cc} x_{k+1} & =\Phi_{k+1|k}x_{k}+U_{k+1|k}{\bar{a}}_{k}+\eta_{k} \end{array}$$

(20) $$\begin{array}{cc} y_{k} & =Hx_{k}+\epsilon_{k} \end{array}$$

where $x_{k}={\left[\;s_{k}^{⊤}\;\nu_{k}^{⊤}\;a_{k}^{⊤}\;\right]}^{⊤}$, $s_{k}={\left[\;s_{X,k}\;s_{Y,k}\;s_{Z,k}\;\right]}^{⊤}\in R^{3}$ [m] is the 3D displacement expressed in the L-system, $v_{k}={\left[\;v_{X,k}\;v_{Y,k}\;v_{Z,k}\;\right]}^{⊤}$ are the corresponding velocities, and $a_{k}={\left[\;a_{X,k}\;a_{Y,k}\;a_{Z,k}\;\right]}^{⊤}$ are the corresponding local accelerations in the L-system,

(21) $$\begin{array}{cc} \Phi_{k+1|k} & =\left[\begin{array}{ccc} I_{3} & TI_{3} & \alpha_{k}^{-2}\left(\alpha_{k}T-I_{3}+e^{-\alpha_{k}T}\right)\\ 0 & I_{3} & \alpha_{k}^{-1}\left(I_{3}-e^{-\alpha_{k}T}\right)\\ 0 & 0 & e^{-\alpha_{k}T} \end{array}\right]\\ U_{k+1|k} & =\left[\begin{array}{c} \alpha_{k}^{-1}\left(-T+\frac{\alpha_{k}T^{2}}{2}+\alpha_{k}^{-1}\left(I_{3}-e^{-\alpha_{k}T}\right)\right)\\ \alpha_{k}^{-1}\left(\alpha_{k}T-I_{3}+e^{-\alpha_{k}T}\right)\\ I_{3}-e^{-\alpha_{k}T} \end{array}\right]\\ H & =\left[\begin{array}{ccc} 0 & 0 & I_{3} \end{array}\right] \end{array}$$

where we recall that *T* is the IMU sampling time, $\alpha_{k}>0$ is a diagonal matrix of dimension 3, and it represents a parameter whose value will be discussed later; $\eta_{k}\in R^{9}$ is white Gaussian noise with zero mean and covariance matrix

(22) $$\Sigma_{k}=\left[\begin{array}{ccc} Q_{11,k} & Q_{12,k} & Q_{13,k}\\ Q_{12,k} & Q_{22,k} & Q_{23,k}\\ Q_{13,k} & Q_{23,k} & Q_{33,k} \end{array}\right]$$

where

$$\begin{array}{c} Q_{11,k}=\frac{1}{2}\Lambda_{k}\alpha_{k}^{-5}\left(I_{3}-e^{-2\alpha_{k}T}+2\alpha_{k}T+\frac{2\alpha_{k}^{3}T^{3}}{3}-2\alpha_{k}^{2}T^{2}-4\alpha_{k}Te^{-\alpha_{k}T}\right)\\ Q_{12,k}=\frac{1}{2}\Lambda_{k}\alpha_{k}^{-4}\left(e^{-2\alpha_{k}T}+I_{3}-2e^{-\alpha_{k}T}+2\alpha_{k}Te^{-\alpha_{k}T}-2\alpha_{k}T+\alpha_{k}^{2}T^{2}\right)\\ Q_{13,k}=\frac{1}{2}\Lambda_{k}\alpha_{k}^{-3}\left(I_{3}-e^{-2\alpha_{k}T}-2\alpha_{k}Te^{-\alpha_{k}T}\right)\\ Q_{22,k}=\frac{1}{2}\Lambda_{k}\alpha_{k}^{-3}\left(4e^{-\alpha_{k}T}-3I_{3}-e^{-2\alpha_{k}T}+2\alpha_{k}T\right)\\ Q_{23,k}=\frac{1}{2}\Lambda_{k}\alpha_{k}^{-2}\left(e^{-2\alpha_{k}T}+I_{3}-2\alpha_{k}T\right)\\ Q_{33,k}=\frac{1}{2}\Lambda_{k}\alpha_{k}^{-1}\left(I_{3}-e^{-2\alpha_{k}T}\right) \end{array}$$

and $\Lambda_{k}>0$ is a diagonal parameter matrix of dimension 3 whose value will be discussed later; $\epsilon_{k}\in R^{3}$ is white Gaussian noise with unknown mean $r_{k}$ and unknown covariance matrix $R_{k}$; ${\bar{a}}_{k}$ is the average value of the maneuvering acceleration over a window of length *N*

(23) $${\bar{a}}_{k}=\frac{1}{N}\sum_{i=k-N}^{k-1}a_{i|i}$$

and $a_{i|i}$ is the estimate of $a_{i}$ at time *i*. Notice that, in the transient initial steps, i.e., for *k* such that 1<k<N, we have ${\bar{a}}_{k}=\frac{1}{k}\sum_{i=0}^{k-1}a_{i|i}$.

The aim of the state space model (19) and (20) is to provide an estimate $x_{k|k}={\left[\;s_{k|k}^{⊤}\;\nu_{k|k}^{⊤}\;a_{k|k}^{⊤}\;\right]}^{⊤}$ of $x_{k}$ such that the estimate of the displacement $s_{k|k}$ is accurate. The latter will be accurate if the estimate $a_{k|k}$ of $a_{k}$ is accurate. From (19), it is not difficult to see that $a_{k+1|k+1}$ is computed according to the following a priori information about the evolution of $a_{k}$:

(24) $$a_{k+1}=\exp\left(-\alpha_{k}T\right)a_{k}+\left(1-\exp\left(-\alpha_{k}T\right)\right){\bar{a}}_{k}+\eta_{a,k},$$

that is, the accelerations are a convex combination of their previous value and their average value (on a window of length *N*). The parameter matrix $\alpha_{k}>0$ tunes the influence of $a_{k}$ and ${\bar{a}}_{k}$ on $a_{k+1}$: if the pedestrian displacement changes slowly over time, then, the diagonal elements of $\alpha_{k}$ should be taken as very large. Notice that $\Lambda_{k}$ tunes how much the prior in (24) should influence the estimate of the accelerations. The choice of the parameters $\alpha_{k-1}$ and $\Lambda_{k-1}$ can be computed by means of the Yule–Walker algorithm, see [76,77] for more details, or in general, a spectral estimation method which estimates an autoregressive process of order one through a moment matching approach [78,79,80,81]. Here, the moments are the covariance lags of order zero and one obtained from the time series $\left\{\;a_{i|i},\;i=k-N\ldots k-1\;\right\}$. Then, we can use the adaptive Kalman filter [76] to compute $x_{k|k}$ and the parameters characterizing the noise process $\epsilon_{k}$.

Once $s_{k|k}={\left[\;s_{X,k|k}\;s_{Y,k|k}\;s_{Z,k|k}\;\right]}^{⊤}$ is computed, then, the estimate $p_{k|k}={\left[\;p_{N,k|k}\;p_{E,k|k}\;\right]}^{⊤}$ of $p_{k}$ can be computed from $s_{k|k}$, $p_{k-1|k-1}$ and $w_{m,k}$ (angular velocity measured from the IMU unit) as follows:

(25) $$\begin{array}{cc} p_{N,k|k} & =p_{N,k-1|k-1}+d_{k}\cos\left(\psi_{k}\right) \end{array}$$

(26) $$\begin{array}{cc} p_{E,k|k} & =p_{E,k-1|k-1}+d_{k}\sin\left(\psi_{k}\right) \end{array}$$

where

$$d_{k}=\sqrt{s_{X,k|k}^{2}+s_{Y,k|k}^{2}}.$$

**Figure 2:**
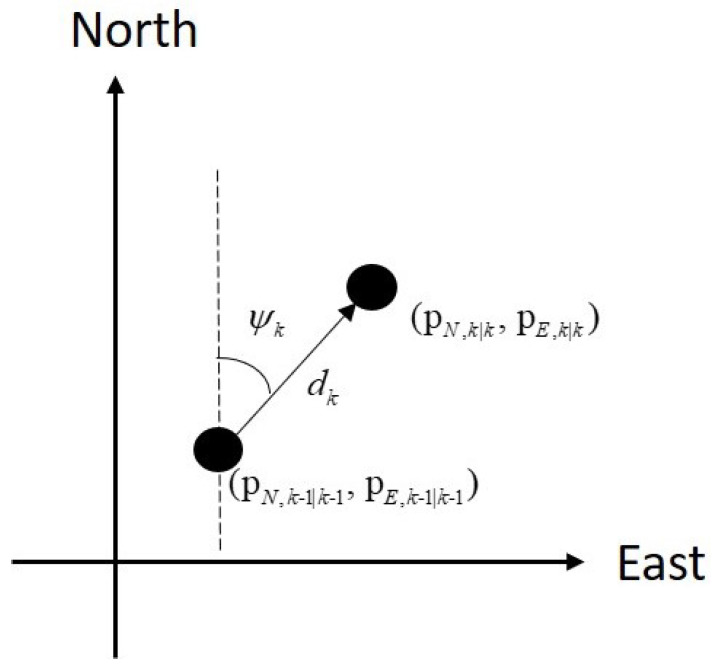
Trajectory generation using the distance covered $d_{k}$ and the yaw angle $\psi_{k}$.

In plain words, the estimate of the pedestrian position is obtained updating the previous one: the distance covered is obtained by $s_{k|k}$ while the direction by the yaw angle $\psi_{k}$, [82]. The process of this trajectory generation is illustrated in Figure 2.

The resulting algorithm at time k=1,2,… is the following:

1. Available information:
  $$y_{k},\;x_{k-1|k-1},\;p_{k-1,k-1}\;w_{m,k},\;P_{k-1|k-1},\;r_{k-1},\;R_{k-1}.$$
2. Compute the average value of the maneuvering acceleration ${\bar{a}}_{k}$ as in (23) where $a_{i|i}$ is obtained from $x_{i|i}$.
3. Compute the parameters $\alpha_{k-1}$ and $\Lambda_{k-1}$ (and thus also $\Sigma_{k-1}$) using $a_{i|i}$ with i=k−N…k−1.
4. Compute $x_{k|k-1}$ and $P_{k|k-1}$ as in (8) and (9) where $\Psi_{k-1}$, *B*, and $Q_{k-1}$ are substituted by $\Phi_{k|k-1}$, $U_{k|k-1}$, and $\Sigma_{k-1}$, respectively.
5. Compute $r_{k}$ and $R_{k}$ as in (10)–(12) where *C* is substituted by *H*.
6. Compute $x_{k|k}$ and $P_{k|k}$ as in (13)–(15) where *C* is substituted by *H*.
7. Compute $p_{k|k}$ as in (25) and (26).

It is worth noticing that the initial condition $p_{0|0}$ is obtained by the last estimate of the pedestrian position obtained in the previous navigation task (i.e., the one in Section 2.1).

## 3. Experiments

In this section, we verify the effectiveness and feasibility of the proposed switched approach through both synthetic and real experiments.

### 3.1. Synthetic Experiment

We firstly analyze the impact of the bias and noise affecting acceleration measurements on the accuracy of pedestrian position estimation. Moreover, we also verify the validity of denoising the acceleration measurements through the estimated average bias (as the method proposed in Section 2 does).

**Figure 3:**
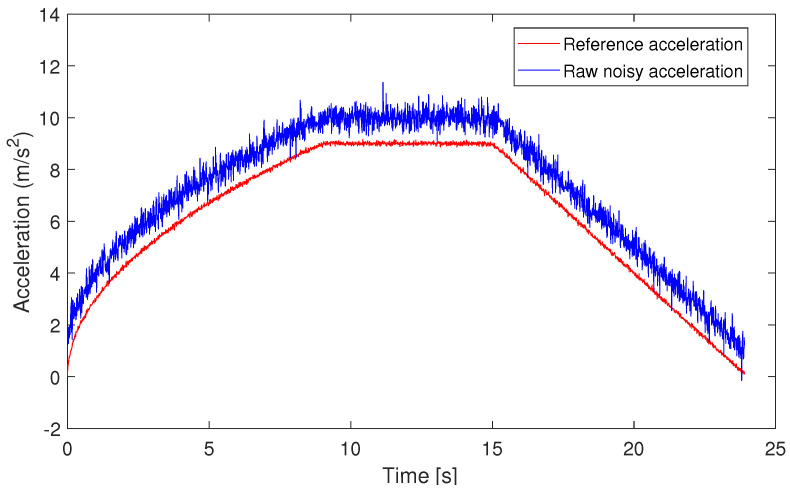
Reference acceleration (red line) and the corresponding measured signal (blue line).

We generate the IMU and GNSS measurements as follows. For simplicity, we only consider the case where the acceleration is different from 0 only on the Y-axis, i.e., it is equal to 0 on the other two axes. We generate the one-dimensional reference acceleration in the L-system as in Figure 3 (red line); the corresponding sampling time is T=0.01 s. This reference describes a situation in which the pedestrian starts to run and then stops. In order to verify the goodness of the estimated average bias on the position estimation, we consider the idealistic setup where the gyro measurements are generated following a Gaussian distribution with zero mean and a small covariance matrix $0.01I_{3}$. Then, we generate the corresponding positions in the ENU-system. The GNSS signals are obtained by corrupting the positions in the ENU-system adding white Gaussian noise with zero mean and covariance matrix $0.005I_{2}$. Since the primary error source in the accelerometer-based pedestrian position estimation is the bias in the form of constant offset and random noise, we generate the corresponding measured acceleration as shown by the blue line in Figure 3, which is generated as the sum of white noise (Gaussian with zero mean and variance equal to 1), the reference acceleration (red line in Figure 3), and a bias (set as 1).

We consider the case where the GNSS signals are available. Thus, we apply the procedure in Section 2.1 to estimate the average bias $\bar{b}$. Here, the forgetting factor is set as ρ=0.1 and N=100. The initial condition are set as follows:

$$x_{0|0}={\left[\;0\;0\;0\;\right]}^{⊤},\;\;P_{0|0}=0.01I,\;\;q_{0}={\left[\;0\;0\;0\;\right]}^{⊤},\;\;r_{0}={\left[\;0\;0\;0\;\right]}^{⊤},\;\;Q_{0}=0.01I,\;\;R_{0}=I.$$

**Figure 4:**
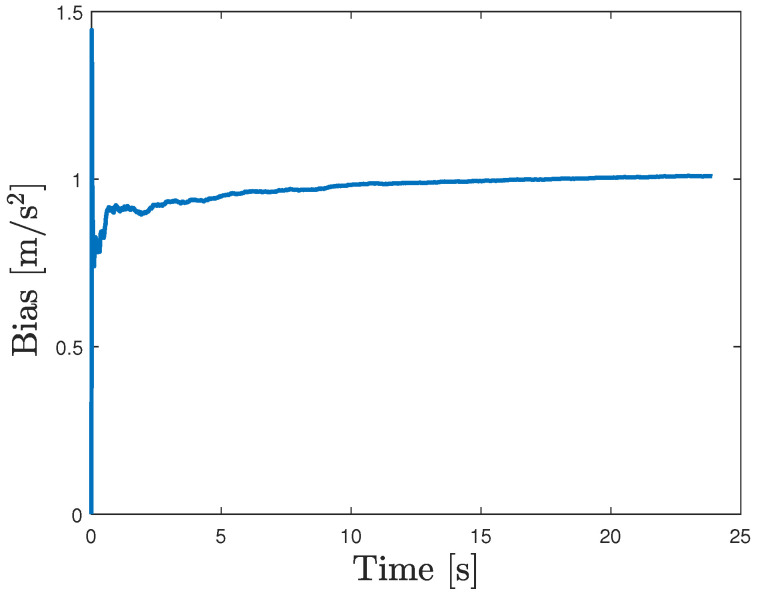
Estimated average bias when its true value is 1.

**Figure 5:**
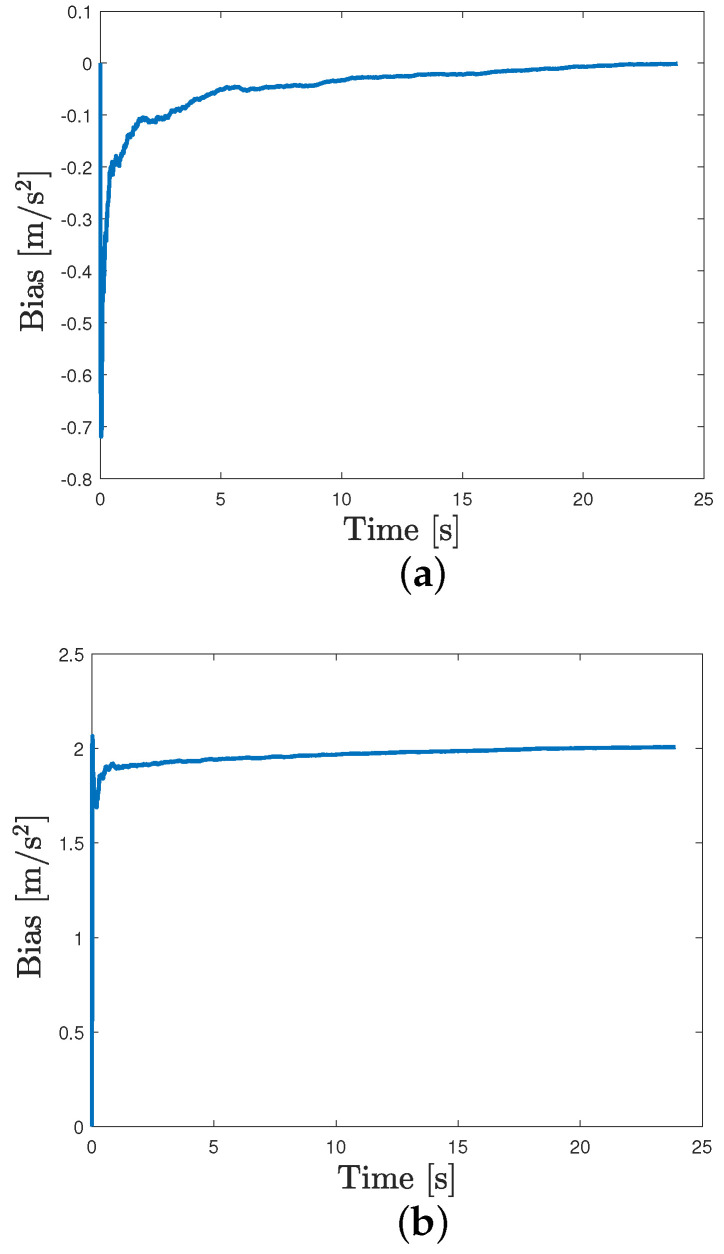
Average bias estimation. (**a**) Estimated average bias when its true value is 0. (**b**) Estimated average bias when its true value is 2.

As shown in Figure 4, the estimated average bias converges to its true value, i.e., 1. Moreover, to further prove its effectiveness, we also set different reference values of bias, i.e., 0 and 2. Figure 5 shows that the average bias can be estimated in a satisfactory way in these cases also.

To further assess the accuracy of the estimated average bias, we use the procedure in Section 2.2, i.e., the one in the case where the GNSS signals are not available, using the IMU signals of before and the bias estimated before (the case in which the true bias is equal to 1). The forgetting factor is set as ρ=0.1, N=100 and the initial conditions as

$$x_{0|0}={\left[\;0\;0\;0\;\right]}^{⊤},\;\;P_{0|0}=0.01I,\;\;r_{0}={\left[\;0\;0\;0\;\right]}^{⊤},\;\;R_{0}=I.$$

**Figure 6:**
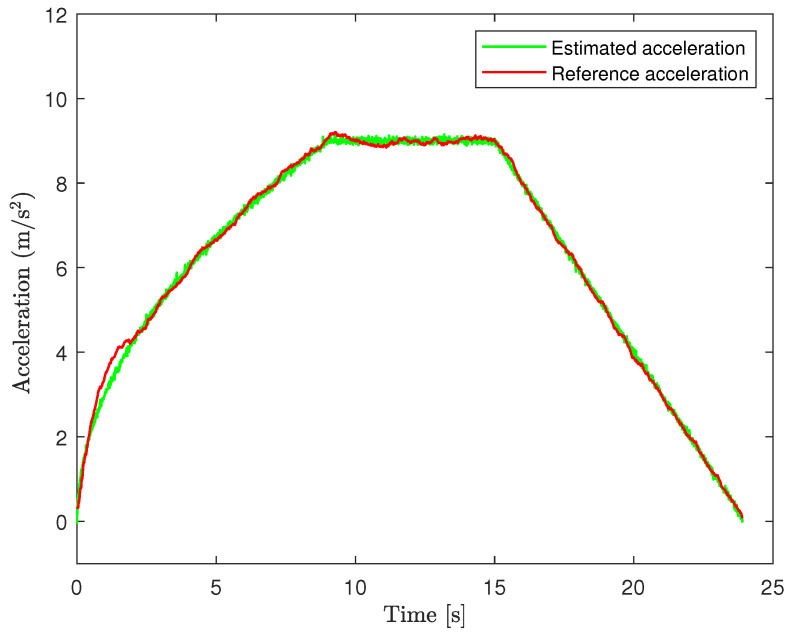
Estimated acceleration and reference acceleration.

**Figure 7:**
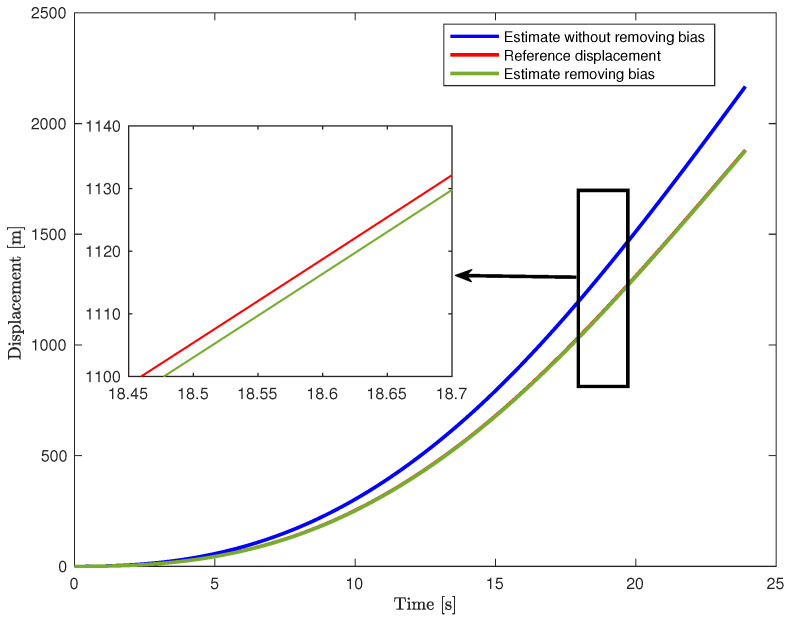
Estimated displacement and reference displacement.

Moreover, we set $p_{0|0}={\left[\;0\;0\;\right]}^{⊤}$. Figure 6 shows that the estimated acceleration on the Y-axis. We notice that the estimate is very accurate. Figure 7 shows the displacement along the Y-axis in the case where the average bias is removed (i.e., our procedure), green line, and not removed, blue line. We observe that our estimate is very accurate. Conversely, if we neglect the influence of the average bias in accelerometer measurements and directly apply the raw acceleration, then, the resulting deviation is significant.

### 3.2. Real Experiment

**Figure 8:**
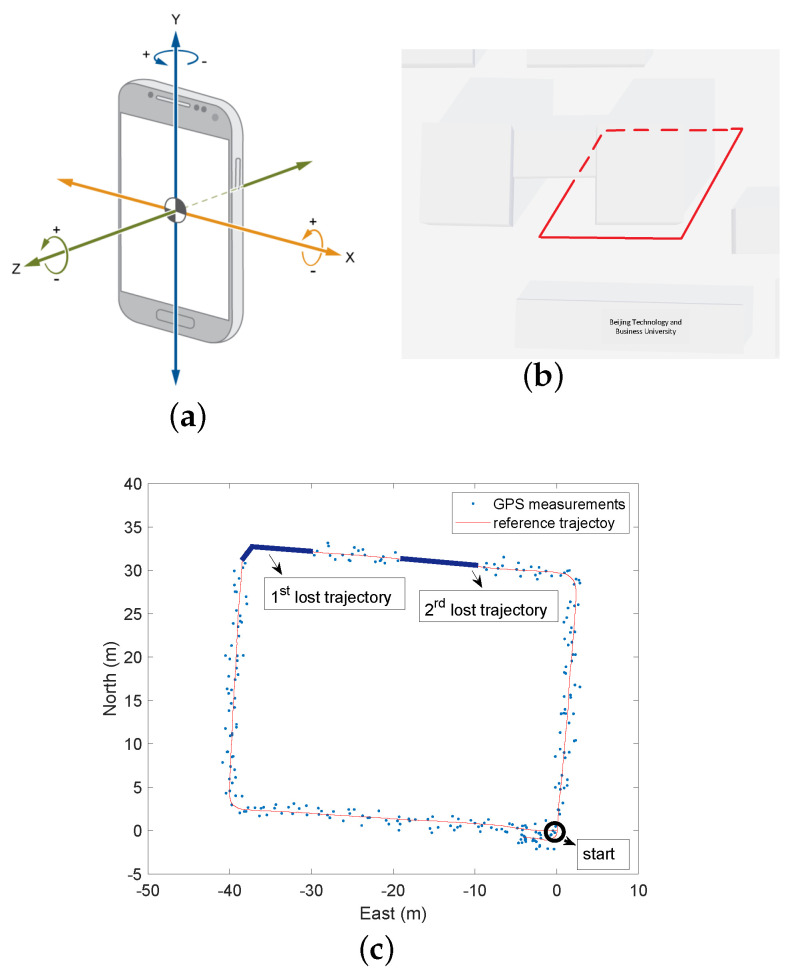
Description of the experiment. (**a**) Axis orientation of the smartphone. (**b**) The pre-planned path. Dashed style means that the pedestrian is walking on an underpass. (**c**) The reference trajectory and GNSS measurements in the ENU-system. The pedestrian started at the black circle, moving clockwise, following the red path.

An outdoor pedestrian navigation experiment was conducted using a smartphone named Huawei Mate 50 (where its axis orientation is illustrated in Figure 8a). The smartphone was held as in Figure 8a and kept as steady as possible by a pedestrian who followed a pre-planned path of approximately 150 m, depicted in Figure 8b. The longitude and latitude of this pre-planned path were sourced from Google Maps. These coordinates were then converted to the ENU coordinate system, represented by the red line in Figure 8c. Moreover, the GNSS raw measurements, i.e., the longitude and latitude of the pedestrian, were collected by the GNSS receiver in the smartphone (Huawei Mate 50) using “MATLAB Mobile”. These coordinates were also converted to the ENU coordinate system, represented by the blue points in Figure 8c, where lost segments are marked using a different line color and style. Hereafter, we shall call these GNSS measurements in the ENU coordinate system as GNSS measurements. Note that, the sampling time of GNSS ($T_{GNSS}=1$ s) is much larger than that of IMU (T=0.01 s). Therefore, we apply the causal zero-order hold interpolation to align the GNSS signals with IMU signals. We estimate the position of the pedestrian using the switched approach of Section 2, leveraging GNSS and IMU signals from the smartphone. Here, the initial conditions as well as the parameters, i.e., ρ and *N*, are set as in Section 3.1. It is worth noticing that the yaw is provided by the IMU, and it is always available. We have found that the raw measurements of the gyro are of reasonable quality under our instrument setups: we only performed a simple online denoising operation (i.e., a low pass causal filtering operation) on these raw measurements.

**Figure 9:**
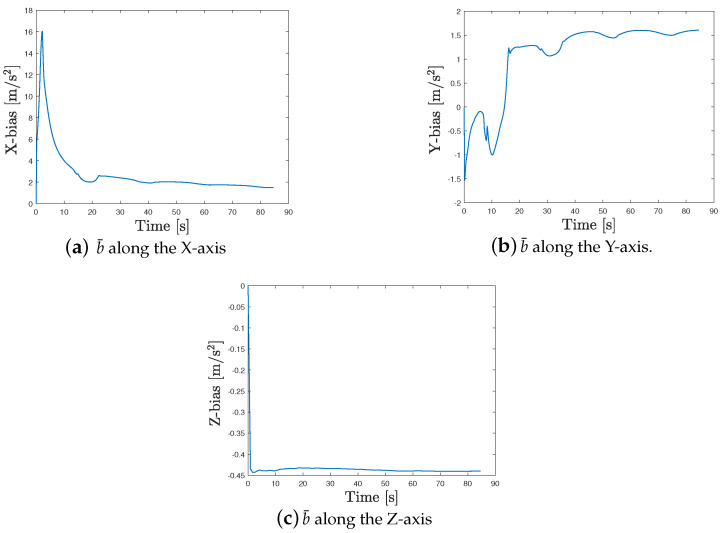
Estimated vector bias $\bar{b}$ in the L-system (first phase).

In the first phase, the GNSS signals are available, and thus, the navigation procedure with GNSS signals of Section 2.1 is applied. Figure 9 shows the estimated average bias $\bar{b}$ during this phase. At the end of this phase, we have $\bar{b}={\left[2.034,1.579,-0.439\right]}^{⊤}$. This is the average bias used in the second phase in which the GNSS signals are not available (it corresponds to the first lost trajectory, see Figure 8c), and thus, the navigation procedure without GNSS signals of Section 2.2 is applied. In the third phase, the GNSS signals are available (it corresponds to the red segment between the first and second lost trajectory, see Figure 8c), and thus, the procedure of Section 2.1 is applied; at the end of this phase, we have $\bar{b}={\left[2.129,1.378,-0.427\right]}^{⊤}$. This is the average bias used in the fourth phase in which the GNSS signals are not available (it corresponds to the second lost trajectory, see Figure 8c), and the navigation procedure of Section 2.2 is used. Finally, in the last phase, the GNSS signals are available, and thus, we apply the procedure of Section 2.1. Note that the initial condition $p_{0|0}$ used in the second phase and the fourth phase are given by the final estimates of the state provided in the first phase and the third phase.

**Figure 10:**
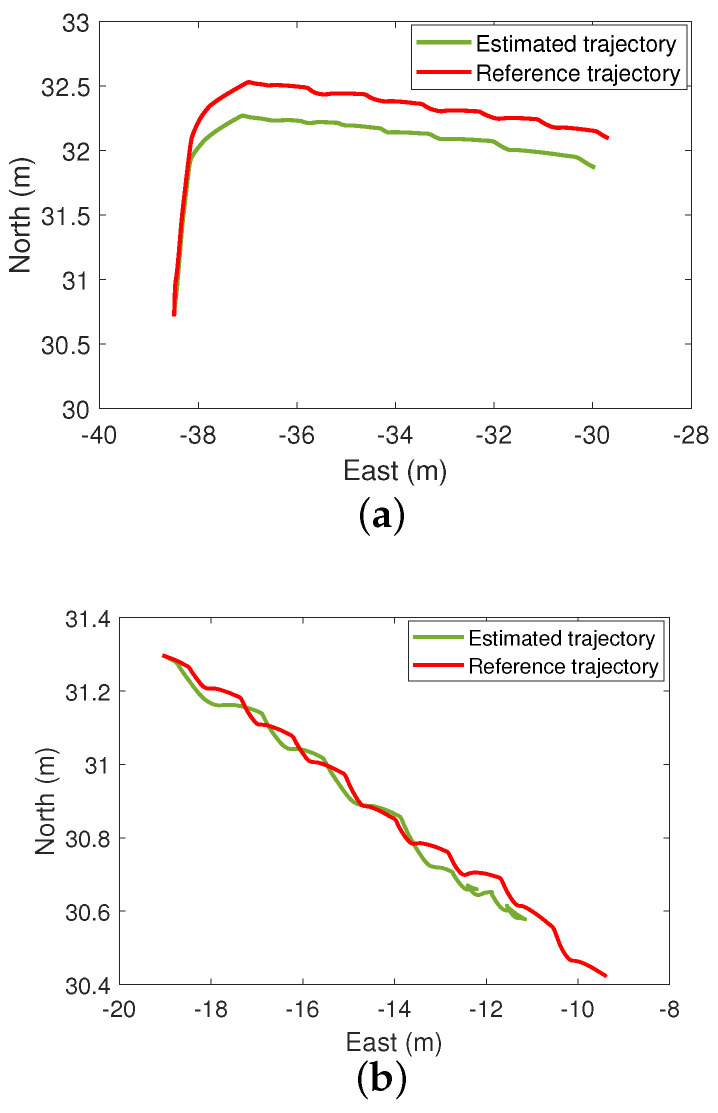
Position estimation in the ENU-system in the GNSS-denied environment. (**a**) Estimated position in the first GNSS-denied trajectory. (**b**) Estimated position in the second GNSS-denied trajectory.

**Figure 11:**
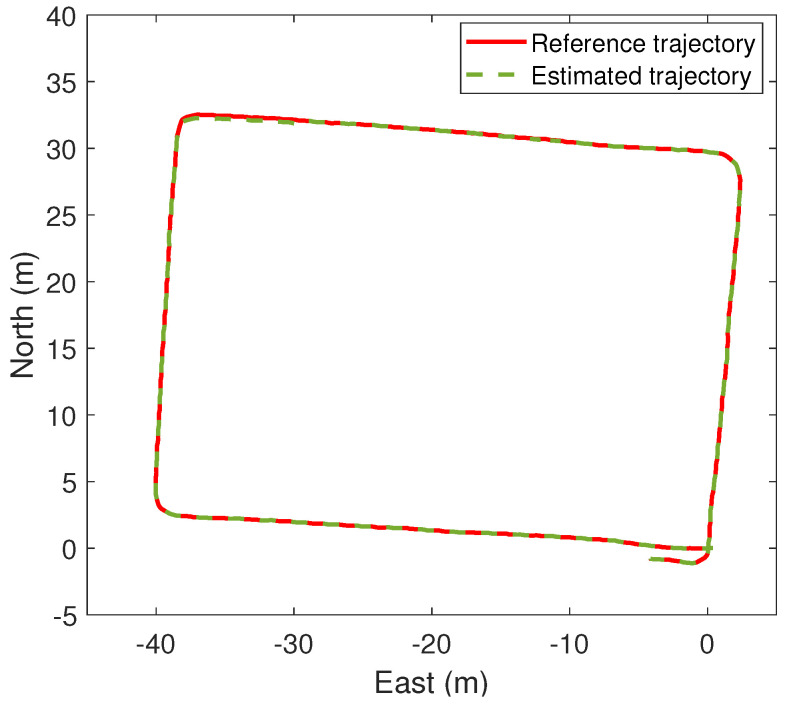
The overall pedestrian position estimation in the ENU-system.

Figure 10 shows the estimated pedestrian position during the first and second time when the GNSS signals are lost (it corresponds to the first and second lost trajectory in Figure 8c, respectively). We can see such an estimate is very accurate even though the GNSS data are not available. As a sanity check, we also estimate the pedestrian position using the IMU signals without $\bar{b}$, and the resulting estimate is highly inaccurate due to cumulative errors. The overall pedestrian position estimates obtained by our method are shown in Figure 11. We see that the accuracy achieved by the proposed algorithm is very good. Finally, Table 1 compares the Root Mean Square Error (RMSE) of the two lost trajectories and the whole trajectory; as we can see, the RMSE is always less than 1 m.

## 4. Conclusions

In this paper, we presented a switched scheme to perform a smartphone-based pedestrian navigation task. The proposed approach estimates in real time the position of the pedestrian also in the case where the GNSS signals are unavailable. More precisely, when GNSS signals are available, the proposed approach estimates both the position and (the average value of) the bias affecting the measurements coming from the accelerometers. This estimated average bias is used to denoise the accelerometer data when the GNSS signals are not available. Unlike the PDR technology, our approach does not require the use of computationally expensive algorithms or invasive sensors, and thus, it can be easily embedded in a smartphone device. Synthetic and real experiments demonstrate the validity and effectiveness of the proposed method in both a GNSS-free environment and GNSS-denied environment.

This study also showed that the estimated average bias contains useful information that can be exploited when the GNSS is not available. So, an interesting question is whether this average bias can be incorporated in the PDR technology, which relies on acceleration measurements coming from the IMU device [83,84], in order to improve the so-called “PDR pedestrian step estimation” task.

## Figures and Tables

**Table 1:** RMSE for the pedestrian position estimation along the east and north directions and in the two-dimensional space.

	East	North	2D
First lost trajectory	0.7146	0.0921	0.7764
Second lost trajectory	0.6909	0.2038	0.8877
Whole trajectory	0.2910	0.0571	0.3135

## Data Availability

Data are contained within the article.
